# Two-dimensional crystallization of monomeric bovine cytochrome *c* oxidase with bound cytochrome *c* in reconstituted lipid membranes

**DOI:** 10.1093/jmicro/dfv381

**Published:** 2016-01-10

**Authors:** Yukiho Osuda, Kyoko Shinzawa-Itoh, Kazutoshi Tani, Shintaro Maeda, Shinya Yoshikawa, Tomitake Tsukihara, Christoph Gerle

**Affiliations:** 1 Picobiology Institute, Department of LifeScience, Graduate School of Life Science, University of Hyogo, 3-2-1 Kouto, Kamighori, Akoh, Hyogo 678-1297, Japan; 2 Cellular and Structural Physiology Institute, Nagoya University, Chikusa-ku, Nagoya 464-8601, Japan; 3 Institute for Protein Research, Osaka University, 3-2 Yamada-oka, Suita, Osaka 565-0871, Japan; 4 Japan Science and Technology Agency(JST), Core Research for Evolutional Science and Technology (CREST), Kawaguchi, Japan

**Keywords:** membrane protein, two-dimensional crystal, electron crystallography, OXPHOS, electron transport chain, mitochondria

## Abstract

Mitochondrial cytochrome *c* oxidase utilizes electrons provided by *c*ytochrome *c* for the active vectorial transport of protons across the inner mitochondrial membrane through the reduction of molecular oxygen to water. Direct structural evidence on the transient cytochrome *c* oxidase–cytochrome *c* complex thus far, however, remains elusive and its physiological relevant oligomeric form is unclear. Here, we report on the 2D crystallization of monomeric bovine cytochrome *c* oxidase with tightly bound cytochrome *c* at a molar ratio of 1:1 in reconstituted lipid membranes at the basic pH of 8.5 and low ionic strength.

Mitochondrial cytochrome *c* oxidase is the terminal oxidase of the respiratory chain and plays a central role in cellular bioenergetics [1]. Utilizing electrons transferred by cytochrome *c* from the bc_1_ complex, cytochrome *c* oxidase couples the reduction of molecular oxygen to water with the pumping of protons across the inner mitochondrial membrane [2]. Both the protons transported to the P-side of the membrane, and the chemical protons from the N-side of the membrane consumed for water formation contribute to the proton motive force across the inner mitochondrial membrane powering ATP formation by the F_o_F_1_ ATP synthase [3,4]. The molecular structure of dimeric bovine cytochrome *c* oxidase has been characterized by X-ray crystallography at high resolution [5, 6, 7] and for several states of the reaction cycle [8].

The transient nature of cytochrome *c* binding to cytochrome *c* oxidase during electron transfer at a turnover rate of more than 600 s^−1^ [9] likely contributes to the difficulty of growing well diffracting 3D crystals of the cytochrome *c* oxidase–cytochrome *c* complex and thus far no 3D crystals of the complex suitable for structure determination by X-ray crystallography have been published for the mitochondrial enzyme. As a consequence, structural evidence based on crystallography for this important interaction is limited to the covalently linked cytochrome *c* domain of the *caa3* type cytochrome *c* oxidase from the thermophilic eubacteria *Thermus thermophilus* [10].

High resolution X-ray structures of bovine cytochrome *c* oxidase reported so far were exclusively of its dimeric form, leaving doubt about the physiological relevance of monomeric cytochrome *c* oxidase in mammalian mitochondria. However, the detection of monomeric cytochrome *c* oxidase in a respiratory supercomplex by single particle cryo-EM [11] has rekindled interest in its oligomeric state.

Electron crystallography of 2D crystals is a powerful tool to investigate the structure of membrane proteins in their physiological environment of the lipid bilayer and at moderate ionic strengths [12–14]. In addition, their two-dimensional geometry also facilitates the addition of water-soluble binding partners such as cytochrome *c* [15]. Earlier electron crystallographic studies of mitochondrial cytochrome *c* oxidase, all conducted before the arrival of X-ray crystal structures, mainly focused on 2D crystals formed by detergent extraction from mitochondrial membranes, i.e. at unknown cytochrome *c* oxidase to cytochrome *c* stoichiometry and without control of the lipid-to-protein ratio (LPR) [16–19]. In contrast, 2D crystallization of the pure, isolated enzyme free from other protein contaminants allows the exact determination of cytochrome *c* oxidase molarity and thus the precise control of cytochrome *c* oxidase/cytochrome *c* stoichiometry and LPR during 2D crystallization.

Here, we describe the 2D crystallization of monomeric bovine cytochrome *c* oxidase with tightly bound cytochrome *c* in reconstituted lipid membranes. The cytochrome *c* oxidase crystalline sample was isolated from bovine heart mitochondria as previously described [20]. Immediately after isolation, cytochrome *c* oxidase was mixed with cytochrome *c* (from horse heart, solubilized in 40 mM Na-Pi, pH 7.8) at a molar ratio of 1:1.1 and incubated on ice for 1 h. Subsequently, the cytochrome *c* oxidase–cytochrome *c* complex was gently mixed with 1-palmitoyl-2-oleoyl-*sn*-glycero-3-phosphocholine (POPC, Nippon Fine Chemical, in 50 mM NaP_i_, pH 7.4, 1 mM DTT, 0.02% NaN_3_ and 1.5% decyl-maltoside) at a LPR of 0.4–0.45. After further incubation on ice for 6 h, aliquots were suspended into dialysis buttons, sealed with dialysis membrane (SpectraPore#7, MWCO 15,000) and dialyzed at 4°C against a buffer (20 mM Tris–HCl, pH 8.5, 2 mM CaCl_2_, 0.02% NaN_3_, 1 mM DTT) containing decyl-maltoside at a concentration of 0.5 of its critical micellular concentration (cmc) for 2 days, at a concentration of 0.25 cmc for one day and detergent free for another 4 days.

Association of cytochrome *c* with the 2D crystals was assessed by comparing absorption spectra of membrane fraction pellet and supernatant after centrifugal spin-down at 18 000*g* for 30 min (Supplementary data online, Fig. 1).

The sample from dialysis buttons (2.5 µl) was applied onto freshly glow-discharged (Eiko IB3 ion coater, at <40 Pa, ∼5 mA for ∼10 s), carbon-coated 400 mesh copper grids (Veco) in the cold-room at 4°C. After brief blotting (Advantec #4), sample was stained using a 2% uranyl acetate solution and air-dried. Images were taken with a JEM1010 transmission electron microscope (JEOL) equipped with a 2:1 tapered 1 × 1 K FastScan F-114T CCD camera (Tietz) at 100 kV and 12 pA cm^−2^, an exposure time of 2 s and a magnification of ×40 000, corresponding to a pixel size of 6 Å. Collected CCD images were transformed to MRC format. All images were processed with the MRC processing package [21] to correct lattice distortions [22].

In this study, the same batches of purified cytochrome *c* oxidase were used for both 3D and 2D crystallization. The fact that the 3D crystals of cytochrome *c* oxidase without cytochrome *c* diffracted to better than 2 Å suggests that the batches are highly purified cytochrome *c* oxidase (Fig. 1). Two-dimensional crystals were abundant and almost all sample observed exhibited ordered arrays (Fig. 2). Under crystallization buffer conditions cytochrome *c* was tightly bound to the 2D crystals, however, raising the ionic strength by dialysis against crystallization buffer containing additional 100 mM KCl released cytochrome *c* from the 2D crystals and impaired their order. Notably, 2D crystallization in the absence of cytochrome *c* resulted in less well-ordered crystals (Supplementary data online, Fig. 2) and at a molar ratio of 1:2 or higher crystals tended to stack in multilayers. This reflects the experience of earlier work by Frey and Murray [19] and possibly suggests conformational stabilization of cytochrome *c* oxidase by cytochrome *c* binding and the existence of a second low affinity binding site [23].

**Fig. 1.**
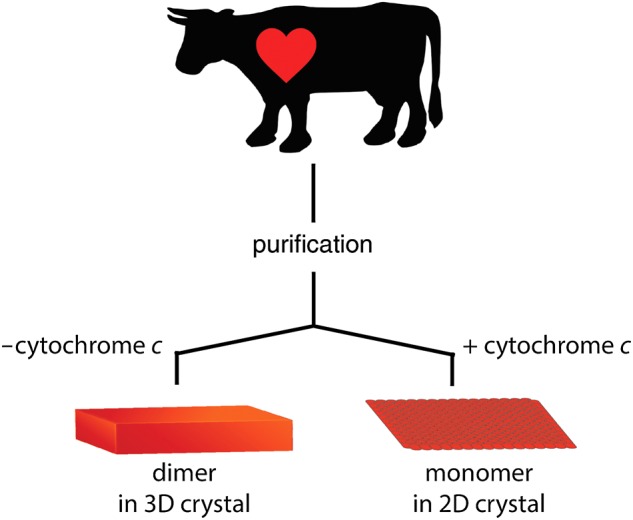
Schematic drawing of the 2D crystallization strategy used in this study. To ensure the highest possible quality of the sample in terms of purity and stability, all 2D crystallization trials were performed exclusively with cytochrome *c* oxidase that yielded 3D crystals diffracting to better than 2 Å.

**Fig. 2.**
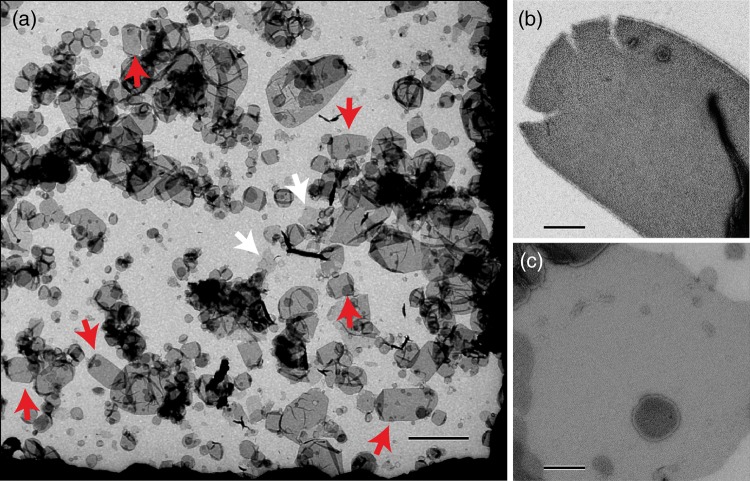
(a) Overview of a typical grid-hole. Almost all membrane sheets showed ordered arrays. The majority of 2D crystals are of vesicular morphology (red arrows) and a far smaller fraction sheet-like (white arrows). Since the sheet-like 2D crystals exhibited sharper diffraction spots, the projection map was calculated solely from these. Scale bar 1 µm. (b) Typical vesicular type 2D crystal. Scale bar 200 nm. (c) Typical sheet-like 2D crystal. Scale bar 200 nm.

The majority of 2D crystals were of vesicular morphology (Fig. 2a), however, sheet-like 2D crystals in negative stain (Fig. 3a) exhibited sharper diffraction patterns (Fig. 3b) and were used for image processing. The projection map calculated at a resolution of 15 Å from six micrographs reveals unit cell dimensions of *a* = 158 Å, *b* = 81 Å and *γ* = 90° containing two monomeric cytochrome *c* oxidase complexes (Fig. 3c, Table 1). Despite the presence of a lipid bilayer, the features of our projection map are very similar to that of previously published ones [17, 19]. In the intermembrane space of respiring mitochondria cytochrome *c* is mainly bound to surface of the inner mitochondrial membrane [24, 25]. The loose packing of the cytochrome *c* oxidase monomer in the 2D crystal would provide sufficient free membrane surface between the complexes for the binding of cytochrome *c* either to the membrane surface alone or lateral to cytochrome *c* oxidase. However, the density distribution in our projection matches the size and shape of one cytochrome *c* oxidase monomer alone (Fig. 3c) and no density suggesting lateral binding of cytochrome *c* to membrane-embedded cytochrome *c* oxidase was detected. Therefore, lateral binding of cytochrome *c* to cytochrome *c* oxidase in the 2D crystal seems unlikely under the current crystallization conditions. Cytochrome *c* oxidase employed in 2D crystallization trials in this study was exclusively from purification batches of cytochrome *c* oxidase, which also yielded 3D crystals of dimeric cytochrome *c* oxidase diffracting to better than 2 Å resolution. Taking this into account with the presence of a lipid bilayer in the 2D crystals and the mild crystallization buffer, it seems very unlikely that the detected oligomeric form of a monomer is an artifact resulting from harsh purification and crystallization conditions and thus should represent a native form of bovine cytochrome *c* oxidase. The presence of monomeric cytochrome *c* oxidase in the 2D crystals is also in line with the recent report of monomeric cytochrome *c* oxidase in a mitochondrial supercomplex [11] comprising Complexes I, III and IV (cytochrome *c* oxidase). Thus in its cellular context of the inner mitochondrial membrane cytochrome *c* oxidase may exist in an equilibrium of both monomeric and dimeric forms.

**Table 1.** Crystallographic table of cytochrome *c* oxidase in complex with cytochrome *c*

*a* = 158 Å, *b* = 81 Å, and *γ* = 90.0°	
Symmetry	*p*1
Resolution	∼1/15 Å^−1^
Overall weighted phase residuals^a^	20.2° (*n* = 6)

^a^Used reflections are better than IQ 5.

**Fig. 3.**
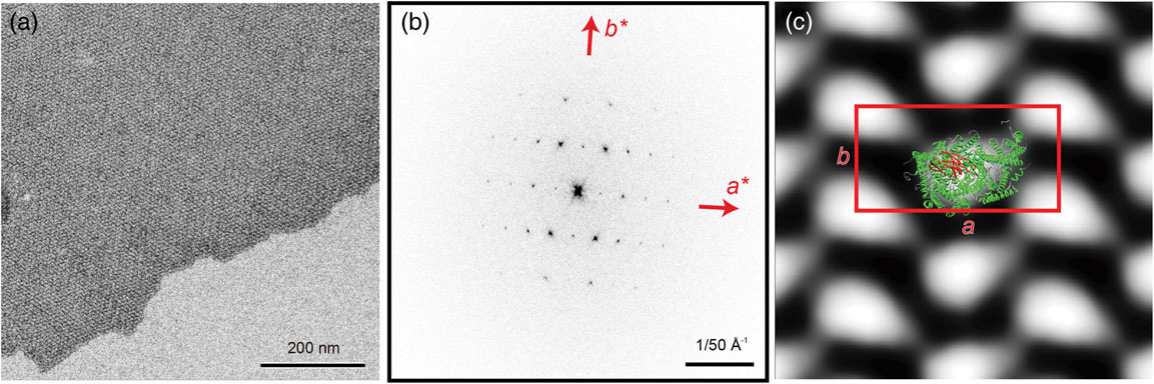
(a) Electron micrograph of a two-dimensional crystal of monomeric bovine cytochrome *c* oxidase with bound cytochrome *c* in negative stain, (b) its Fourier transform and (c) a projection map calculated from six two-dimensional crystals with the unit cell parameters, *a* = 158 Å, *b* = 81 Å and *γ* = 90.0° (outlined in red), and the structure of a bovine cytochrome *c* oxidase monomer (PDB: 1OCC) was manually superimposed. A putatively cytochrome *c* binding beta-sheet is depicted in red.

For further analysis in 3D of the here reported cytochrome *c* oxidase–cytochrome *c* 2D crystals, three different avenues are open. First, the canonical approach of acquiring cryo images from a sufficient number of well diffracting 2D crystals that can be merged. If this, however, proves difficult due to, i.e. persistent heterogeneity in unit cell parameters, alternative ways of data collection and analysis can overcome these challenges. One strategy is to obtain image data from double-axis tilt series taken from a single 2D crystal, which then can be used for 3D reconstructions by electron crystallography as has been performed for 2D crystals of *T. thermophilus* V-ATPase [26] and bovine Complex I [27]. Another approach that can be used for even poorly ordered 2D crystals containing only a few hundred unit cells is to obtain cryo-electron tomograms of a 2D crystal and then treat tomographic volumes of the regions of highest order by electron crystallographic analysis. This approach has been successfully applied to cryo-electron tomograms of poorly ordered vesicular 2D crystals of bovine F_o_F_1_ ATP synthase [28]. The sensitivity of cytochrome *c* binding to changes in ionic buffer strength will make it necessary to mitigate evaporation effects during cryo grid preparation by using the carbon sandwich technique [29, 30]. The here described 2D crystals now set the stage for further structural analysis of bovine cytochrome *c* oxidase in its monomeric and cytochrome *c* bound form and in the physiological environment of the lipid bilayer.

We have used electron crystallography of two-dimensional crystals to show that the monomeric form of mammalian cytochrome *c* oxidase is physiologically relevant and not an artifact of its isolation from mitochondria. This result is supported by the close to physiological environment of the lipid bilayer in the two-dimensional crystals and the mild crystallization conditions in which cytochrome *c* is tightly bound to the cytochrome *c* oxidase at the precise molar ratio of 1:1 between cytochrome *c* oxidase and cytochrome *c* at low ionic strength and a basic pH.

## Supplementary data

Supplementary data are available at http://jmicro.oxfordjournals.org/.

## Funding

This work was supported by the Special Coordination Fund for Promoting Science and Technology of MEXT, Japan (to C.G.), a Platform for Drug Design, Discovery and Development grant from MEXT, Japan (to C.G.), the JST/CREST (to T.T. and C.G.), a Grants-in-Aid for Scientific Research (C) from MEXT, Japan (to K.T.) and a Grants-in-Aid for Scientific Research (A) 2247012 and (B) 26234567 from MEXT, Japan (to S.Y.).
